# Status seizures as a secondary effect from the “squash drinking syndrome” in an infant

**DOI:** 10.1002/jpr3.12043

**Published:** 2024-02-14

**Authors:** Antonio Rosell‐Camps, Lucinda F. Bunce, Bernardí Barceló‐Martí, Artur Sharluyan‐Petrosyan

**Affiliations:** ^1^ Department of Pediatrics, Unit of Gastroenterology, Hepatology y Pediatric Nutrition Son Espases University Hospital Palma de Mallorca Spain; ^2^ Department of Pediatrics Health Research Institute of the Balearic Islands (IdISBa) Palma de Mallorca Balearic Island Spain; ^3^ Department of Pediatrics, Clinical Analysis Service Son Espases University Hospital Palma de Mallorca Balearic Island Spain; ^4^ Department of Pediatrics, Unit of Pediatric Intensive Care Son Espases University Hospital Palma de Mallorca Balearic Island Spain

**Keywords:** hyponatremia, hypotonic drinks, seizure status

## Abstract

**Abstract:**

The “hypotonic drink syndrome” is characterized by loss of appetite, normal activity levels and, in some cases, intestinal disturbances in children with an intake of more than 30% of the recommended daily calories in the form of non‐dairy drinks. Diarrhea and growth retardation are possible complications due to the amount of nonnutritive calorie intake (“empty calories”) contained in this type of hypotonic beverages.

We present the case of an 11‐month‐old boy who suffered a “Squash drinking syndrome” requiring admission to the pediatric intensive care unit because of a status seizure secondary to a severe hyponatremia (118 mmol/L) due to massive ingestion of hypotonic drinks, such as squash. The seizure did not subside until sodium levels reached 123 mmol/L with hypertonic saline (3%). Neurological, renal, digestive, endocrine and metabolic problems were all ruled out and normal sodium levels were maintained with dietary recommendations and a restriction of hypotonic fluid intake.

**Conclusions:**

To prevent these situations it is important to be aware of this entity and to know how to identify the possible complications that may appear after excessive ingestion of hypotonic drinks, as in the case of our patient, ranging from lack of appetite, growth failure and diarrhea, to a status seizure.

## 1 INTRODUCTION

The “squash drink syndrome” was first described in 1995 and has been reported as a cause of inappetence with poor weight gain, normal activity levels, as well as nonspecific diarrhea. It is characterized by a caloric intake of more than 30% of the daily recommendations in hypotonic beverages, such as squash drinks.^1^ These patients show a clinical improvement and complete recovery after the restriction of intake of these hypotonic drinks.

We report the case of an infant who developed a hyponatremic status seizure secondary to a high intake of hypotonic beverages.

## 2 CLINICAL CASE

An 11‐month‐old infant was attended in the emergency department during the summer for irritability, inconsolable crying, limb hypertonia and hypersalivation which began 30 min ago. Subsequently, a loss of consciousness and loss of gaze appeared. Oxygen, rectal diazepam and intravenous midazolam were administered in repeated doses but the seizure did not subside. Intravenous administration of valproic acid and levetiracetam was started, which led to remission of the seizure state, but he relapsed within a few minutes. Therefore, the patient was admitted to the Intensive Care Unit, where a blood test detected sodium values of 118 mmol/L. Hypertonic saline (3%) was administered, achieving complete remission of the status seizure and increasing the plasma sodium to 123 mmol/L. After correcting the hyponatremia levels progressively (Table 1), our patient had no new seizures. We carried out a lumbar puncture with cytochemistry and bacterial culture which was negative for neurotropic viruses. A cranial computed tomography scan, blood and urine toxicology study and renal function assessment were also performed, with normal results.

**Table 1:** Evolution of plasma sodium concentration.

Date	Sodium (mmol/L)
1st day (admission)	118
Remission of the seizure	123
After 2 h	129
After 8 h	139
After 24 h	142
After 36 h	141

We present a case report of a healthy child from the United Kingdom on holiday in Majorca, with a weight of 10 kg (percentile 45) and a height of 70 cm (percentile 12) according to the WHO graph. He had no personal medical history of vomiting, diarrhea, fever, anorexia or failure to thrive. While questioning the patient's parents about his clinical history, we detected that he intakes approximately 10 bottles of 330 mL of blackcurrent squash a day. A sample of the squash drink was analyzed in the hospital laboratory containing 25 mg/L of glucose and undetectable sodium levels. After ruling out other secondary causes, we came to the conclusion that the status epilepticus was possibly secondary to the hyponatremia, caused by the excessive intake of this hypotonic drink.

The patient was breastfed until the age of 2 months, and then he was fed with a baby formula until he was 6 months old. Since then, solid food was introduced and dairy products were replaced for diluted squash drinks (drinking between 3 and 3.5 L a day (314 mL/kg/d)). Blackcurrent squash is a hypotonic drink that you dilute in water, being very popular in Anglo‐Saxon countries. Up to a 70% of preschool children and a 50% of infant school groups never drink plain water.^2^

During the stay of our patient in the hospital, the fluid restriction and the urine output led to the maintenance of normal plasma sodium levels without needing supplements. Neurological, renal, digestive, endocrine and metabolic problems were all ruled out. The patient was discharged from the hospital 72 h after admission with dietary recommendations.

## 3 DISCUSSION

The most frequent causes of a status seizures in childhood (from 1 to 5 years) in Pediatric Intensive Units (PIU) are epileptic syndromes, febrile convulsions and encephalopathies.^3^ However, it can also be presented in patients with electrolyte imbalance such as hyponatremia. This hyponatremia is usually due to infectious alterations (acute gastroenteritis) or a lesser extent to endocrine causes (adrenal insufficiency, inadequate secretion of ADH), renal (renal insufficiency), psychiatric (potomania) or sweat loss (cystic fibrosis).^4^ Our case does not belong to any of these subgroups because the status seizure was due to incorrect infant feeding practices.

The excessive intake of hypotonic drinks or fruit juices can result in the appearance off a diarrhea with loss of nutrients, lack of appetite and may lead to a failure to thrive due to the amount of nonnutritional energy, also known as “empty calories” provided in these types of drinks. In a study by Petter et al.^2^ they described that a 70% of preschool children did not drink plain water, being replaced by squash, fruit juices, and so forth. In our patient, the excessive intake of hypotonic drinks led to a status seizure that required admission to ICU. Other cases of seizures secondary to hyponatremia after ingesting 2 L of squash^5^ without reaching status seizure have already been reported. The detection of the cause of a hyponatremia without diarrhea, vomiting or hormonal alterations is challenging for a health care practitioner. Therefore, an extensive anamnesis including all the dietary information of each patient is critically important for the diagnosis.^6^

To prevent these situations, it is important to know the possible complications that may appear after an excessive intake of squash, ranging from a lack of appetite with failure to thrive and diarrhea, to a status epilepticus. Good and basic nutritional education is the key to make these families aware of the possible consequences that drinking high amounts of hypotonic and soft drinks instead of dairy and plain water may lead to.

## CONFLICT OF INTEREST STATEMENT

The authors declare no conflict of interest.

## ETHICS STATEMENT

Informed patient consent was obtained for publication of the case details.

## Supporting information

Supporting information.
