# Supplementary material for: Status seizures as a secondary effect from the “squash drinking syndrome” in an infant
Source: JPGN Rep. 2024 Feb 14;5(2):228–30. doi: 10.1002/jpr3.12043 (PMC11093902; doi:10.1002/jpr3.12043)
Supplement: Supplementary file 1 — Supporting information. [file JPR3-5-228-s001.docx]

**APPENDIX**

**Table 1. Evolution of plasma sodium levels**

| **POINT OF TIME** | **SODIUM LEVELS (mmol/L)** |
| --- | --- |
| Hospital admission | 118 |
| Remission of the seizure | 123 |
| After 2 hours | 129 |
| After 8 hours | 139 |
| After 24 hours | 142 |
| After 36 hours | 141 |
